# Concurrent Infection with Murine Typhus and Scrub Typhus in Southern Laos—the Mixed and the Unmixed

**DOI:** 10.1371/journal.pntd.0002163

**Published:** 2013-08-29

**Authors:** Koukeo Phommasone, Daniel H. Paris, Tippawan Anantatat, Josée Castonguay-Vanier, Sommay Keomany, Phoutthalavanh Souvannasing, Stuart D. Blacksell, Mayfong Mayxay, Paul N. Newton

**Affiliations:** 1 Lao-Oxford-Mahosot Hospital-Wellcome Trust Research Unit (LOMWRU), Microbiology Laboratory, Mahosot Hospital, Vientiane, Lao PDR; 2 Centre for Tropical Medicine, Nuffield Department of Clinical Medicine, Churchill Hospital, University of Oxford, Oxford, United Kingdom; 3 Faculty of Tropical Medicine, Mahidol University, Bangkok, Thailand; 4 Salavan Provincial Hospital, Salavan, Salavan Province, Lao PDR; 5 Faculty of Postgraduate Studies, University of Health Sciences, Vientiane, Lao PDR; University of California San Diego School of Medicine, United States of America

Scrub typhus, murine typhus, and spotted fever group rickettsia all occur in the Lao PDR (Laos) [1], [2]. Scrub typhus and murine typhus account for ∼16% and 10%, respectively, of acute undifferentiated fever in blood culture–negative adults admitted to hospital in the capital city, Vientiane [1]. However, typhus-like illnesses are significant diagnostic challenges; patients with leptospirosis, dengue, typhoid, and malaria are also common and can present with similar symptoms and signs. Although these pathogens are common and mixed (or concurrent) infections are expected, the laboratory diagnosis of mixed infection is a vexed subject. Reports of mixed infections often use only serological criteria. The problems of antibody persistence and interspecies cross-reaction raise uncertainty as to whether these results represent true mixed infections, sequential infections, or cross-reactions. We report a patient with concurrent scrub typhus and murine typhus, demonstrated by dual PCR positivity, and discuss evidence for identifying mixed infections.

## Patient

As part of a study investigating the aetiology of fever among patients with negative malaria tests, we recruited patients at Salavan Provincial Hospital, Salavan Province, southern Laos [3]. A 20-year-old female rice farmer from Naxay Village (15°62′37.06″N; 106°33′42.13″E), Salavan District, whose house was surrounded by vegetable gardens, presented at Salavan Provincial Hospital in July 2009 with 14 days of headache associated with three days of fever, myalgia, and vomiting, having taken five days of oral cephalexin. She was febrile (38.5°C), but physical examination was otherwise normal without rash or eschar. She was suspected to have scrub typhus and was prescribed empirical doxycycline and amoxicillin for seven days and recovered fully. Ethical approval was granted by the Lao National Ethics Committee for Health Research and the Oxford Tropical Research Ethics Committee, United Kingdom, and the patient provided written consent to publication of clinical details.

Subsequently, the patient's acute serum sample was assayed for immunoglobulin (Ig)M and IgG antibody titres against reference *O. tsutsugamushi* antigens (pooled Karp, Kato, and Gilliam) and *R. typhi* antigen (Wilmington strain) by indirect immunofluorescent assay [4]. The admission serum had titres of scrub typhus IgM<400 and IgG = 1,600 and murine typhus IgM<400 and IgG<400. Convalescent serum was not available. DNA from admission EDTA anticoagulated buffy coat was extracted and used as template for the *O. tsutsugamushi* 47-kDa-gene-based real-time PCR assay, the *R. typhi ompB*-gene-based real-time PCR assay, the *Rickettsia* genus 17-kDa-gene-based real-time PCR assay, and the *O. tsutsugamushi groEL*-gene-based real-time PCR. Each run contained duplicate low-positive dilutions of linearized pGEM plasmids, ranging from 10^4^ to a single copy/µl, as external controls (Table 1).

**Table 1:** Overview of PCR-based and DNA sequencing results.

PCR positivity criteria			
Scrub typhus	Murine typhus	Strength of evidence	Technique [reference]
47-kDa real-time PCR (2× pos.)	17-kDa real-time PCR (2× pos.)	Strong*	Jiang *et al.*, 2004 [20]
*groEL* real-time PCR (2× pos.)	*ompB* real-time PCR (2× pos.)	Strong*	Henry *et al.*, 2007 [21]Paris *et al.*, 2009 [22]
56-kDa nested PCR (620 bp)47-kDa nested PCR (785 bp)	17-kDa nested PCR (524 bp)	Very strong. Product size confirmation via gel electrophoresis	Horinouchi *et al.*, 1996 [23]Jiang *et al.*, 2012 [24]
DNA sequences for 47-kDa^1^ and 56-kDa^2^ nested PCR amplicons	DNA sequence for 17-kDa nested PCR amplicon^3^	Extremely strong. BLAST result with 97–100% coverage for amplicon similarities	Altschul *et al.*, 1990 [25]

* positivity criteria in analogy to the Minimum Information for Publication of Quantitative Real-Time PCR Experiments (MIQE) [19]. GenBank accession numbers:

1 BankIt1587796 Seq2 KC283067.

2 BankIt1587796 Seq3 KC283068.

3 BankIt1587796 Seq1 KC283066.

The buffy coat was positive for the *O. tsutsugamushi* 47-kDa and *groEL* target genes as well as the *Rickettsia* genus 17-kDa and *R. typhi ompB* target genes by the diagnostic real-time PCR assays, indicating potential dual positivity for *O. tsutsugamushi* and *Rickettsia* spp. The copy numbers determined for both pathogens were within the range normally seen at our laboratory (56/59 and 75/130 copies/µl for the 47-kDa and *ompB* real-time assays, respectively). That samples were processed in separate pre- and post-PCR work areas, the evidence of multigene PCR positivity, and that no other dual positive samples were found makes contamination extremely unlikely. Further characterisation was performed (Table 1), including a panel of conventional nested PCR assays targeting the 17-kDa (product size 524 bp), 56-kDa (product size 620 bp), and 47-kDa (product size 785 bp) target genes. All three assays provided positive PCR amplicons and the products were purified and sequenced by Macrogen (Korea). Among the candidates with the same BLAST score results for the 17-kDa PCR amplicon (367 bp sequence), the geographically closest related strain found was *R. typhi* strain TH1526 (max. score 640, max. identity 99%, query coverage 97%, E-value 3e-180), from a patient with murine typhus from Chiang Rai, N. Thailand. The 47-kDa amplicon (744 bp) matched *O. tsutsugamushi* Ikeda strain (max. score 1314, query coverage 100%, E-value 0.0) and the nested 56-kDa amplicon (523 bp) matched *O. tsutsugamushi* T1125175_KH 56-kDa type-specific antigen (max. score = 640, query coverage = 99%, E-value 0.0).

The infecting *O. tsutsugamushi* strain is very similar to the human-pathogenic Cambodian isolate T1125175_KH and the animal-derived (*Rattus rajah*) Thai strain TA763, making this the first Lao scrub typhus patient with a strain similar to another nonhuman vertebrate strain [5], [6]. Similarly, human pathogenicity of a Kato-related TA716-like *O. tsutsugamushi* strain originally described from the Indochinese ground squirrel (*Menetes berdmorei*) has been recently reported from Thailand [7].

## Mixed Infections

We present a patient with clear molecular diagnostic evidence of concurrent mixed infection with scrub typhus and murine typhus. Such infections may go unrecognized. Although clinically similar, the diseases have markedly different pathophysiology [8]. Although both pathogens would be expected to respond to doxycycline, *O. tsutsugamushi* generally causes the more severe disease and would not be expected to respond to fluoroquinolones, which have been used for murine typhus [9]. Mixed infection with these two pathogens was demonstrated using PCR and IFA among three patients in Yunnan Province, China [10].

Although culture or molecular detection should be the gold standard for demonstrating mixed infection with very high specificity, this approach will suffer from low sensitivity, as significant proportions of patients with good evidence of mono-infection (with fourfold rises in specific IgM) are PCR negative for both scrub typhus [11] and murine typhus (unpublished data). Moreover, there are cross-reactions between IgM against *O. tsutsugamushi* and *R. typhi* [12] and very few objective data on serological responses in confirmed mixed infections. Western blotting has been used to distinguish serological responses [13]. In Vientiane City, 4% of well adults had IgG antibodies against both scrub typhus and murine typhus [2], suggesting the possibility of previous exposures to both organisms and/or serological cross-reactions.

Mixed *O. tsutsugamushi* and *Leptospira* spp. infections have been reported, but none of these included positive PCR or culture for both pathogens (Table 2). Such infections are especially important as leptospirosis would be expected to respond to penicillins or cephalosporins while scrub typhus would not [14]. Mixed Q fever and scrub typhus infections have been reported in Taiwan but only using serological assays. Mixed infections of *Plasmodium falciparum* with both scrub typhus and murine typhus diagnosed by PCR and/or dynamic serology was documented among febrile pregnant women on the Thai–Burmese border (Table 2). Interpretation would be more intricate if either (or both) pathogen(s) caused chronic infections. This has not been demonstrated for *R. typhi* (although we can find no evidence that it has been expressly looked for), but there have been suggestions that *O. tsutsugamushi* may cause long-term infections [15], [16].

**Table 2:** Reports of apparent mixed infections in Asia that included rickettsioses.

Rickettsial pathogen and diagnosis	Additional pathogen and diagnosis	Number of patients*Evidence grade*	CountryReference
*O. tsutsugamushi*Positive dot blot immunoassay (correlates with IgG titres ≥1∶1,600 or IgM titres ≥1∶400)	*Leptospira* spp.MAT 4-fold rise in titre or single titre ≥1∶320	9/22 (41%) of patients with leptospirosis had evidence for scrub typhus*Grade III*	NE ThailandWatt *et al.*, 2003 [26]
*O. tsutsugamushi*IgM single titre ≥1∶80	*Leptospira* spp.MAT single titre of 1∶400	1 patient with cholecystitis, pancreatitis, and acute renal failure*Grade III*	TaiwanWang *et al.*, 2003 [27]
*O. tsutsugamushi*4-fold rise in specific IgG or IgM titre to ≥1∶200 by IFA or a single titre of ≥1∶400	*Leptospira* spp.Culture, MAT, IFA. 4-fold rise in specific IgG or IgM titre to ≥1∶200 by IFA or a single titre of ≥1∶400	62/540 (12%) of patients with leptospirosis had evidence for scrub typhus*Grade II*	NE ThailandSuputtamongkol *et al.*, 2004 [28]
*O. tsutsugamushi*Admission IFA IgM titre 1∶80 & IgG 1∶40	*Leptospira* sp.MAT 4-fold rise in antibody titre and *Burkholderia pseudomallei* by blood culture	1 patient with melioidosis had evidence of leptospirosis and scrub typhus*Grade II* (meliodosis+leptospirosis)*Grade III* (scrub typhus+melioidosis)*Grade III* (scrub typhus+leptospirosis)	TaiwanLu *et al.*, 2005 [29]
*O. tsutsugamushi*Serology technique not statedPatient 1: PCR positivePatient 2: PCR and IgM & IgG positivePatient 3: PCR positive, IgM positive, and 4-fold rise in IgGPatient 4: PCR positive, IgM positive, and 4-fold rise in IgG	*Leptospira* spp.Serology technique not statedPatient 1: single titre 1∶1,600Patient 2: single titre 1∶800Patient 3: antibody titre increased >4-fold risePatient 4: antibody titre increased >4-fold rise	4 patients with leptospirosis had evidence for scrub typhus*Grade II*	TaiwanHo *et al.*, 2006 [30]
*O. tsutsugamushi*Admission IFA IgM ≥1∶80 plus 4-fold rise in IgG titre on paired sera	*Leptospira* spp.MAT seroconversion to 1∶400	1 patient with acute renal failure and pulmonary haemorrhage*Grade II*	TaiwanChen *et al.*, 2007 [31]
*O. tsutsugamushi*IFA 4-fold rise in titre or a single IgM titre ≥1∶80	*Leptospira* spp.MAT 4-fold rise in titre or a single titre ≥1∶320	7/87 (8%) of patients with leptospirosis or scrub typhus had evidence for both pathogens*Grade II*	TaiwanLee *et al.*, 2007 [32]
*O. tsutsugamushi* & *R. typhi*IFA 4-fold rise or a single titre of ≥1∶400	*Leptospira* spp.Culture or MAT 4-fold rise or a single titre of ≥1∶400	11/296 (4%) of patients with leptospirosis had evidence for infection with scrub typhus or murine typhus*Grade II*	NE ThailandPhimda *et al.*, 2007 [33]
*O. tsutsugamushi*PCR positive and ≥4-fold rise in IgG	*R. typhi*PCR positive and ≥4-fold rise in IgG	3/8 (38%) of febrile farmers PCR positive for scrub typhus or murine typhus were PCR positive for both*Grade I*	ChinaZhang *et al.*, 2007 [10]
*O. tsutsugamushi* & *R. typhi*IFA IgM ≥1∶80 or 4-fold rise in IgG	*Coxiella burnetii*IFA anti-phase II IgG ≥1∶320 or IgM ≥1∶80 or a 4-fold rise in IgG titre	5/144 (3%) of patients with Q fever or typhus (scrub and murine) had evidence for both infections*Grade II*	TaiwanLai *et al.*, 2009 [34]
*O. tsutsugamushi* & *R. typhi*PCR, culture or IFA 4-fold rise in IgM or IgG	*Plasmodium falciparum*Giemsa malaria films	5/51 (10%) of pregnant women with malaria had evidence for murine typhus or scrub typhus*Grade I* for scrub typhus-malaria and *grade II* for murine typhus-malaria	NW ThailandMcGready *et al.*, 2010 [35]
*O. tsutsugamushi*IFA seroconversion to IgG 1∶320 & IgM 1∶160	*Leptospira* spp. MAT seroconversion to 1∶1,600	1 patient with shock and respiratory failure*Grade II*	TaiwanWei *et al.*, 2012 [36]

We suggest that reports of mixed infections include an explicit discussion of the likely specificity and sensitivity of the diagnostic assays used and the likelihood that the observations represent true concurrent mixed infections (or coinfections), or sequential infections due to persistence of antibody or false positives due to assay cross-reactions (“dual positivity”). A grading system of evidence, analogous to the GRADE guidelines and Infectious Diseases Society of America guidelines [17], [18], may be helpful. For example, grade I (culture or molecular detection of both pathogens or direct observation such as in a malaria film), grade II (serological diagnosis with either seroconversion or fourfold antibody responses to both pathogens, without evidence of cross-reactions, or using Western blotting), and grade III (serological diagnosis based on admission serology without exclusion of cross-reactions or antibody persistence or culture, molecular, or admission serological detection). Grades I to III would have decreasing specificity but increasing sensitivity in diagnosing true mixed infections. Seroconversion could also be regarded as grade I evidence if documented with a diagnostic test providing highly specific evidence for seroconversion. The relative importance of sensitivity and specificity will depend on the question being asked and the clinical use of the data. When different grades of evidence are used for different pathogens in a “mixed” infection, we suggest that the grade with the highest number (least specificity) is used.

For patients with grade I evidence, further care is required as molecular methods have different specificities for pathogen diagnosis. Real-time PCR specificity is higher if type-specific genes are used (e.g., 56-kDa and 47-kDa genes for *O. tsutsugamushi*) than if genus-specific genes are used (17-kDa genes for *Rickettsia* spp.), which again are stronger than nonspecific conserved “housekeeping” genes (e.g., *groEL* and 16S rRNA). Sequencing should be attempted if conventional (nested) PCR products are obtained, as BLAST analysis will provide high-level confidence with confirmation of the amplicon similarity to gene sequences deposited in GenBank and/or genotyping using SNPs will allow for discrimination at a more subtle level.

We suggest that where possible mixed infections should be confirmed by culture or detection of specific nucleic acid sequences and that the introduction of a grading system for the strength of evidence for mixed infections should be considered.
